# Study of the mechanism by which Xiaoyan decoction combined with E7449 regulates tumorigenesis in lung adenocarcinoma

**DOI:** 10.1111/jcmm.18467

**Published:** 2024-06-19

**Authors:** Xu Zheng, Yanyan Han, Lili Gu, Shan Gao, Yan Lv, Chong Li

**Affiliations:** ^1^ First Teaching Hospital of Tianjin University of Traditional Chinese Medicine Tianjin China; ^2^ National Clinical Research Center for Chinese Medicine Acupuncture and Moxibustion Tianjin China

**Keywords:** E7449, lung adenocarcinoma, tankyrase, Wnt/β‐catenin signalling, Xiaoyan decoction

## Abstract

TNKS is a new target for the treatment of lung adenocarcinoma, the synergistic effects of the TCM compound Xiaoyan decoction and the TNKS inhibitor E7449 in the intervention on TNKS were investigated, and the possible underlying mechanisms involved were clarified. Immunohistochemistry was used to analyse TNKS expression in tumour tissues. The impact of targeting TNKS on cell growth, invasion, apoptosis, key genes and signalling pathways was investigated in tumour cells by Western blotting, rescue experiments, colony formation assays, flow cytometry and label‐free experiments. Tumour xenografts with A549 cells were then transplanted for in vivo study. We found that TNKS high expression was closely related to the advanced tumour stage and tumour size in lung adenocarcinom. After TNKS was knocked down in vitro, the growth, proliferation, migration and invasion were markedly reduced in A549 and H1975 cells. We subsequently applied the Xiaoyan decoction and TNKS inhibitors to intervene in lung adenocarcinoma. Xiaoyan decoction and E7449 suppressed TNKS expression and inhibited adenocarcinoma cell proliferation, migration, invasion and apoptosis in vitro. Proteomic analysis revealed that E7449 treatment may be most closely associated with the classic Wnt/β‐catenin pathway, whereas Xiaoyan decoction treatment may be related to the WNT/PLAN pathway. Xenograft studies confirmed that E7449 or Xiaoyan decoction inhibited lung tumour growth in vivo and attenuated the Wnt signalling pathway in adenocarcinoma. These findings suggest that TNKS is a novel therapeutic target. TCM preparations and small molecule inhibitors are expected to constitute an effective combination strategy.

## 1 INTRODUCTION

Lung cancer is a common malignant tumour, and its morbidity and mortality rank first among malignant tumours.^1^ As a common histological type of lung cancer, lung adenocarcinoma accounts for approximately 50% of lung cancers, and the overall survival rate is low.^2^ TNKS was initially discovered to bind with telomerase repeat binding sequences and belongs to the family of proteins responsible for poly ADP‐ribosylation, which includes TNKS1 and TNKS2.^3^ TNKS1 has been shown to be upregulated in a variety of cancer types, including breast, colon and bladder cancer.^4^, ^5^, ^6^ At present, TNKS inhibitors are widely used. Nan Li^7^ reported that AMP‐activated protein kinase (AMPK) activation by LKB1 is regulated by TNKS. LKB1 activation by TNKS inhibitors induces AMPK activation and suppresses tumorigenesis. Guo et al.^8^ reported that XAV939 decreased the viability of the NCI‐H446 small cell lung cancer (SCLC) cell line by inducing cell apoptosis through the Wnt signalling pathway. A phase 1 clinical trial revealed that E7449 demonstrated good antitumour activity and tolerability in the treatment of patients with advanced solid tumours.^9^ Traditional Chinese medicine has found good economic value and few antitumor side effects. Xiaoyan decoction has been applied in the clinic for more than 20 years in China. Xiaoyan decoction has detoxification‐strengthening activity and strong antitumor activity, and has shown good efficacy in the clinical treatment of lung adenocarcinoma for many years.^10^, ^11^ A study revealed that Xiaoyan decoction may be effective for treating patients with advanced non‐small cell lung cancer. The total remission rate significantly increased compared to that with other treatments, reducing the incidence of adverse reactions related to liver and kidney dysfunction, gastrointestinal tract abnormalities, and bone marrow suppression. Ultimately, this treatment yields a satisfactory prognosis for advanced non‐small cell lung cancer patients.^12^ At present, the study of the antitumour mechanism of TCM compounds is a popular topic of research.^13^, ^14^, ^15^ However, the specific mechanism by which Xiaoyan decoction regulates tumour cells has seldom been reported. Our previous study showed that the expression of the TNKS protein was significantly downregulated after Xiaoyan decoction treatment of lung adenocarcinoma cells. E7449, a TNKS small molecule inhibitor, can also reduce TNKS expression in lung adenocarcinoma cells. Both drugs target TNKS to exert their effects. On this basis, the anticancer effect and possible mechanism of action of the TNKS small molecule inhibitor and traditional Chinese medicine Xiaoyan decoction on lung adenocarcinoma cells were evaluated.

In this study, we investigated the relationship between TNKS and clinicopathological features and verified the biological function of TNKS in A549 and H1975 cell lines. Lung adenocarcinoma A549 cells were subsequently used as the experimental cell line to explore the effects of Xiaoyan decoction alone or in combination with E7449 on the biological behaviour of lung adenocarcinoma cells mediated by the Wnt pathway, and the specific mechanism of action was assessed through multiple intervention experiments and analysis of multiple indicators.

## 2 MATERIALS AND METHODS

### 2.1 Reagents

The TCM Xiaoyan decoction (preparation no. 40019) is an internal preparation of the First Affiliated Hospital of Tianjin University of Chinese Medicine, that consists of *Astragalus mongholicus* Bunge, *Salvia miltiorrhiza* Bunge, *Prunella vulgaris* L., *Curcuma longa* L. and *Scleromitrion diffusum* (Willd.) R.J.Wang. The plant name was checked with data from http://www.worldfloraonline.org/ mentioning the data of accessing that website. After strict reflux extraction, the final concentration was 0.3 g/mL. The preparation was centrifuged at 10000 *r*/min for 3 min, sterilized with a 0.22‐μm filter and diluted with medium to a 100 g/L concentration. HPLC (Figure S1) revealed that the main components of the Xiaoyan decoction were Pseudostellariae cyclic peptide B, curcumin, ursolic acid and astragaloside. The TNKS small molecule inhibitor E7449 (10 mol/L) was purchased from Selleck.cn (USA).

The primary antibodies used were mouse anti‐TNKS (cat no. ab13587; Abcam, Cambridge, UK), mouse anti‐β‐catenin (cat no. AF6266; Affinity Biosciences), mouse anti‐Myc proto‐oncogene protein (c‐Myc) (cat no. sc‐40; Santa Cruz Biotechnology, Inc. Dallas, TX, USA), rabbit anti‐bcl‐2 (cat no. AF6139; Affinity Biosciences), rabbit anti‐caspase‐3 (cat no. AF6311; Affinity Biosciences), TNKS polyclonal antibody (cat no.18030‐1‐AP, proteintech), rabbit polyclonal anti‐PKA alpha/beta/gamma CAT (cat no. AF7746; Affinity Biosciences), rabbit polyclonal antibody to Duplin (CHD8) (cat no. DF9383; Affinity Biosciences), rabbit polyclonal anti‐stbm (VANGL1) (cat no. DF4607; Affinity Biosciences), rabbit polyclonal anti‐IDUNA (RNF146) (cat no. Bs‐11669R; Bioss), AXIN1 Polyclonal antibody (cat no.16541‐1‐AP, proteintech) and mouse anti‐β‐actin (cat no. MAB8929; OriGene Technologies, Inc.).

### 2.2 Patient samples

A total of 74 lung adenocarcinoma tissues and corresponding adjacent tissues were collected from the Department of Pathology at the First Teaching Hospital of Tianjin University of Traditional Chinese Medicine (Tianjin, China). All of the patients were diagnosed with primary lung adenocarcinoma between January 2016 and December 2019, and did not receive preoperative chemotherapy, radiotherapy, immunotherapy or targeted therapy. All clinical data were collected. The research approach was approved by the Clinical Research Ethics Committee of the First Teaching Hospital of Tianjin University of Traditional Chinese Medicine. All patients provided written informed consent. Sections from all cases were reviewed and confirmed to be single‐subtype alveolar lung adenocarcinoma by two senior pathologists affiliated with the First Teaching Hospital of Tianjin University of Traditional Chinese Medicine. The use of the tissue samples for this study was approved by the First Teaching Hospital of Tianjin University of Traditional Chinese Medicine Medical Ethics Committee (Tianjin, China) (ethical lot number: TYLL2018 [K] character005).

### 2.3 Immunohistochemistry

The tissue wax blocks were serially sectioned at 3 μm, dewaxed in xylene, hydrated with gradient ethanol, 3% hydrogen peroxide solution was used to block the endogenous peroxidation enzyme, washed with PBS buffer and subsequently heated with EDTA buffer, pH 9.0 for 2 min to perform antigen retrieval. The sections were incubated in 3% H_2_O_2_ for 15 min at 25°C and then incubated with mouse anti‐TNKS monoclonal antibody (cat no. ab13587; Abcam, Cambridge, UK) (1:200 dilution) overnight at 4°C. The slides were then incubated with horseradish peroxidase‐conjugated goat anti‐rabbit/mouse secondary antibodies (cat no. PV9000; OriGene Technology, Inc.) at 37°C for 30 min. Samples known to express TNKS served as a positive control, whereas PBS was used instead of the primary antibody as a negative control. Positive samples were obtained from the Department of Pathology, First Teaching Hospital of Tianjin University of Traditional Chinese Medicine. The intensity of staining was evaluated using the following criteria: 0, negative; 1, light brown; 2, medium brown; and 3, dark brown. The extent of staining was scored as follows: 0, 0% of cells stained; 1, 1%–25% stained; 2, 26%–50% stained; 3, 51%–75% stained; and 4, 76%–100% stained. The final score was calculated by multiplying the intensity score by the extent score. The staining results were divided into two categories according to the final score: ≤6, low expression and ≥8, high expression.

### 2.4 Real‐time PCR

TRIzol reagent was used for RNA extraction. RNA concentrations were measured with an ultramicrofluorescence spectrophotometer. Then, reverse transcription was implemented. Real‐time PCR (RT‐PCR) was performed using an UltraSYBR mixture (Cwbiotech) and the mRNA was analysed using a PCR instrument (BIO‐RAD96). The results were normalized using glyceraldehyde‐3‐phosphate dehydrogenase (GAPDH) as an internal control. The primers used for real‐time PCR are shown in Table S1. Gene expression was analysed using the 2^−ΔΔCt^ method.

### 2.5 Cell culture and transfection

Four human lung adenocarcinoma cell lines (SK‐LU‐1, CALU, A549 and H1975) and a human normal bronchial epithelial cell line (BEAS‐2B) were obtained from the Chinese Academy of Sciences Cell Bank (CASCB, China). The cell lines were cultured in RPMI‐DMEM supplemented with 10% foetal bovine serum, 100 U/mL penicillin and 100 μg/mL streptomycin at 37°C in a humidified incubator containing 5% CO_2_. TNKS short hairpin RNA (shRNA; Syngenta, China) (shRNA‐TNKS) and the corresponding control RNA (shRNA NC), TNKS overexpression (OE‐TNKS) lentiviral vector and empty TNKS vector (Syngenta, China) (OE‐NC) were transfected into cells in logarithmic growth phase. The sequences of the transfected TNKS overexpression plasmid and shRNA oligonucleotides are shown in Table S2.

### 2.6 CCK‐8 assay

The cells were subjected to routine digestion, and cell concentrations were adjusted to inoculation at 5 × 10^3^ per well in 96‐well plates (80 μL per well). Twenty microliters of CCK‐8 solution was added to each well for 24 h, 48 h, 72 h and 96 h, and the plates were incubated at 37°C for another 4 h. Finally, the absorbance value (A value) of each well was measured on a microplate reader (k = 490 nm).

### 2.7 Wound healing assay

Marker lines were drawn with the diameter of the marker through the hole on the back of the 24‐well plate. Cells with good growth status were seeded in 24‐well plates. The cells were left perpendicular to the marker line scratch with a 200 μ L pipette tiphead and washed with PBS solution three times. Photographs were taken at 0 h and 48 h. The distances were measured by Image‐Pro Plus 6.0 software.

### 2.8 Transwell invasion assay

Cell suspensions were prepared and the cell concentration was adjusted to 1 × 10^5^. Matrigel (1:3 dilution) was added to 4°C precooled serum‐free DMEM medium to a final concentration of 1 mg/mL. Two hundred microlitres cell suspension was added to the upper chamber of the Transwell plate, and 500 μL of DMEM was added to the lower chamber. After 48 h, the liquid in the upper chamber was aspirated, the chamber was removed, the cells were fixed with methanol solution for 30 min at room temperature, and stained with crystal violet. Three fields were randomly taken under an inverted microscope and the number of perforating cells was calculated.

### 2.9 Colony formation assay

The transfected cells were counted at 400 cells seeded in 6‐well plates.

The cells were added to the DMEM medium containing a volume fraction of 10% FBS and cultured at a temperature of 37°C. The medium was changed once every 2 or 3 days, and the colony formation was observed after 2 weeks.

### 2.10 PARylation assay of TNKS

The transfected cells were spread in 6‐well plates at 105 cells/mL. Cells were grouped as A549‐OE‐TNKS, A549‐OE‐NC, H1975‐OE‐TNKS, H1975‐OE‐NC; A549‐shRNA‐TNKS01, A549‐shRNA‐NC, H1975‐shRNA‐TNKS01, H1975‐shRNA‐NC. After 48 h, cells were digested, the precipitate was collected by centrifugation and the supernatant was discarded. After added 300 μL PBS of each tube, cells were broken by repeated freeze–thaw with liquid nitrogen. Then, the protein suspension was obtained by centrifugation at 12000 rpm for 10 min. The followed experiment carried out according to the instructions of the TNKS PARylation assay kit (BPS Bioscience, #78405‐1).

### 2.11 Western blot analysis

A549 cells were spread in 6‐well plates at 10^5^ cells/mL. The E7449 group received 10 mol/L of the small molecule inhibitor E7449. A final concentration of 100 g/L Xiaoyan decoction was given to the Xiaoyan decoction group. E7449 and Xiaoyan decoction were administered at the same time in the Xiaoyan decoction combined with E7449 group. Western blotting was also conducted as previously described.^16^, ^17^ After 24 h, the samples were homogenized in RIPA lysis buffer (Beyotime Biotechnology, Shanghai, China), supplemented with a cocktail of protease and phosphatase inhibitors, and incubated on ice for 30 min. And the protein concentration was determined using the bicinchoninic acid (BCA) protein detection system. Then, 10 μg of cell lysate was denatured for 5 min at 95°C and separated via 10% SDS PAGE. Proteins were transferred from the gels onto a PVDF membrane and blocked with 5% BSA in TBST (Tris‐buffered saline with 0.1% Tween‐20) for 1 h. The samples were incubated overnight with primary antibodies at 4°C. Primary antibodies were diluted with 3% dried skim milk in TBST solution. The primary antibodies used were TNKS polyclonal antibody (cat no.18030‐1‐AP, Proteintech, 1:1000), anti‐PKA (cat no. AF7746; Affinity Biosciences, 1:1000), anti‐Duplin (CHD8) (cat no. DF9383; Affinity Biosciences, 1:1000), anti‐stbm (VANGL1) (cat no. DF4607; Affinity Biosciences, 1:1000), anti‐β‐catenin (cat no. AF6266; Affinity Biosciences, 1:1000), anti‐Myc proto‐oncogene protein (c‐Myc) (cat no. sc‐40; Santa Cruz Biotechnology, 1:1000), anti‐bcl‐2 (cat no. AF6139; Affinity Biosciences, 1:1000), anti‐caspase‐3 (cat no. AF6311; Affinity Biosciences, 1:1000), rabbit polyclonal antibody to IDUNA (RNF146) (cat no. Bs‐11669R; Bioss, 1:1000), AXIN1 Polyclonal antibody (cat no.16541‐1‐AP, proteintech, 1:1000) and mouse anti‐β‐actin (cat no. MAB8929; OriGene Technologies, Inc., 1:5000). After washing with TBST three times, the membranes were incubated with secondary antibodies for 2 h at 25°C. The protein samples were visualized using enhanced chemiluminescence substrate and imaged with C‐DiGit 3600 (Li‐Cor, USA).

### 2.12 Label‐free protein quantification

A549 cells treated with 9% NaCl (as a control group), Xiaoyan decoction, E7449 or Xiaoyan unit E7449 for 48 h were harvested, and proteomic changes were assessed by label‐free proteomics.

Proteins with more than two peptides and fold‐changes >1.25 or <0.8 were regarded as differentially expressed proteins (DEPs) (*p* < 0.05).

The protein was first extracted, digested and desalinated. Then, LC–MS/MS analysis was performed. Finally, the functional analysis of the proteins and the identification of DEPs were performed. Gene Ontology (GO) analyses were also conducted using the InterProScan 5 program against the protein database. Clusters of Orthologous Groups (COG) and Kyoto Encyclopedia of Genes and Genomes (KEGG) were used to analyse the protein expression and pathways.

### 2.13 In vivo tumorigenesis assay

The experiments were carried out in accordance with the guidelines issued by the National Institutes of Health Guide for the Care and Use of Laboratory Animals (NIH Publications no. 8023, revised 1978). Eight‐week‐old BALB/c male nude mice were purchased from Shanghai SLAC Laboratory Animals Co., Ltd. (Shanghai, China). The biological behaviours of tumour formation, invasion and metastasis were observed by subcutaneous xenograft mouse model. First, 5 × 10^6^ A549 cells were injected subcutaneously into the groin of BALB/c nude mice (*n* = 6 per group). Tumour dimensions were measured by digital callipers every 3 days. Dosing was started when the tumour size was 50–100 mm^3^. Weight, tumour diameter and tumour volume were measured before administration and at 3, 7, 10 and 14 days after administration. All groups of mice were administered by gavage. The concentration of NaCl administered to the control group was 0.9%, 2 mL/bid. The concentration of E7449 administered in the E7449 group was 100 mg/kg, qd. The administered concentration of Xiaoyan decoction in the group was 1.365 g/kg, bid. The concentration of E7449 administered was 100 mg/kg, qd and the administered concentration of Xiaoyan decoction was 1.365 g/kg, bid.in the combination group. The mice were sacrificed by cervical dislocation 14 days later, and the tumours were excised for weighing. The tumour volume was calculated with the formula *V* = (length × width^2^)/2. The tumour tissues were fixed with formaldehyde and stained with haematoxylin and eosin. In addition, part of the tumour tissue was subjected to WB detection.

### 2.14 Transmission electron microscope image capture

To morphologically confirm the structure of the tumour cells and mitochondria, ultrastructural analysis was conducted. According to the electron microscope sampling method, the tumour tissue was exfoliated, the size of 1mm^3^ sample was taken, and tissue was rapidly prefixed with the electron microscope solution glutaraldehyde. The buffer was changed once every 2 h for a total of three times. Then, the tumour tissue was fixed with osmic acid, dehydrated with gradient ethanolacetone, embedded in epoxy resin, sliced by ultrathin microtome and observed under a transmission electron microscope.

### 2.15 Statistical analysis

All of the experiments were repeated at least three times. The results are shown as the mean ± standard deviation. The Chi‐square test was used to evaluate the association between TNKS expression and the clinical characteristics of lung cancer patients. Student's *t*‐test and one‐way ANOVA were used to analyse the differences between the two groups.

A p value <0.05 was considered to indicate statistical significance. All the calculations were performed using GraphPad Software.

## 3 RESULTS

### 3.1 TNKS was upregulated in lung adenocarcinoma cells and tissues and correlated with tumour size and tumour‐node‐metastasis stage

**FIGURE 1:**
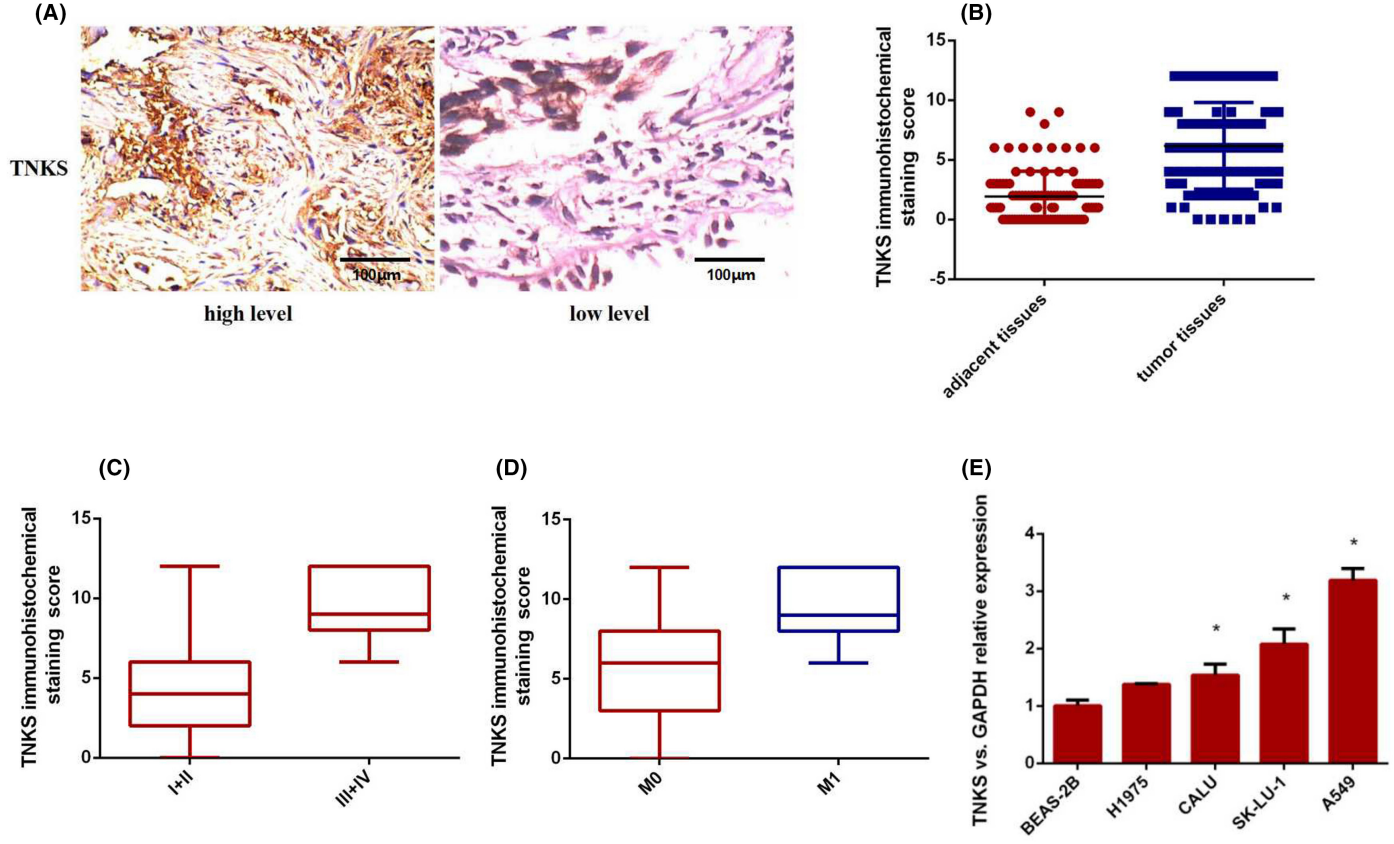
TNKS expression and its relationship with clinicopathological characteristics. (A) High and low expression of TNKS determined by immunohistochemistry. (B) TNKS expression in lung cancer tissues compared with that in adjacent normal tissues. (C, D) Positive TNKS expression was detected more frequently in advanced and metastatic lung adenocarcinoma patients than in patients with early and non‐metastatic lung cancer. (E) TNKS expression in human normal lung bronchial epithelial cells (BEAS‐2B) and four lung cancer cell lines (CALU, SK‐LU‐1, A549, H1975).

To confirm the expression of TNKS in lung cancer tissues, we examined TNKS immunohistochemistry (IHC) expression in 74 pairs of and found that the expression of TNKS was significantly more highly expressed in lung cancer tissues than in adjacent normal tissues (Figure 1A,B). To further investigate the relationship between TNKS expression and clinicopathological features, 74 human lung cancer tissue samples were divided into two subgroups based on the IHC staining score indicating TNKS expression. (≤6, low expression; ≥8, high expression). Correlation regression analysis revealed that the high expression of TNKS in 74 cases of lung adenocarcinoma was closely correlated with tumour size (*p* = 0.000) and tumour‐node‐metastasis (TNM) stage (*p* = 0.001). There were no correlation between TNKS expression and gender (*p* = 0.414), age (*p* = 0.597), smoking history (*p* = 0.915) or differentiation (*p* = 0.105) (Table 1). Furthermore, TNKS was more frequently expressed in patients with advanced and metastatic lung adenocarcinoma than in patients with early and non‐metastatic lung cancer (Figure 1C,D). And then, TNKS expression in human normal lung bronchial epithelial cells (BEAS‐2B) and four lung cancer cell lines (CALU, SK‐LU‐1, A549, H1975) was determined via RT–qPCR. As shown in Figure 1E, TNKS expression in all four cell lines was significantly higher than that in normal bronchial epithelial cells. Of the four cell lines, the relative expression of TNKS was lowest in H1975 cells and highest in A549 cells. For this reason, we chose the A549 and H1975 cell lines as he follow‐up subjects. In conclusion, the results suggested that TNKS was abnormally upregulated in lung cancer tissues and cells and was closely related to tumour size and TNM stage, indicating that high expression of TNKS may play an oncogenic role in lung adenocarcinoma.

**TABLE 1:** TNKS expression and the association with clinicopathological characteristics.

Characteristics	*n*	TNKS level		*x* ^2^	*p*‐Value
		High	Low		
Total cases	74				
Gender					
Male	47	21	25	0.668	0.414
Female	27	15	12		
Age (years)					
≥60	62	31	31	0.279	0.597
<60	12	5	7		
Tumour size (cm)					
≤3	45	13	32	17.947	0
>3	29	23	6		
Smoking history					
Yes	46	22	24	0.011	0.915
No	28	14	14		
Differentiation					
High and moderate	43	15	28	2.626	0.105
Poor	31	21	10		
TNM stage					
I + II	47	10	37	10.112	0.001
III + IV	27	23	4		

### 3.2 TNKS promoted the tumorigenic properties of lung adenocarcinoma cells

**FIGURE 2:**
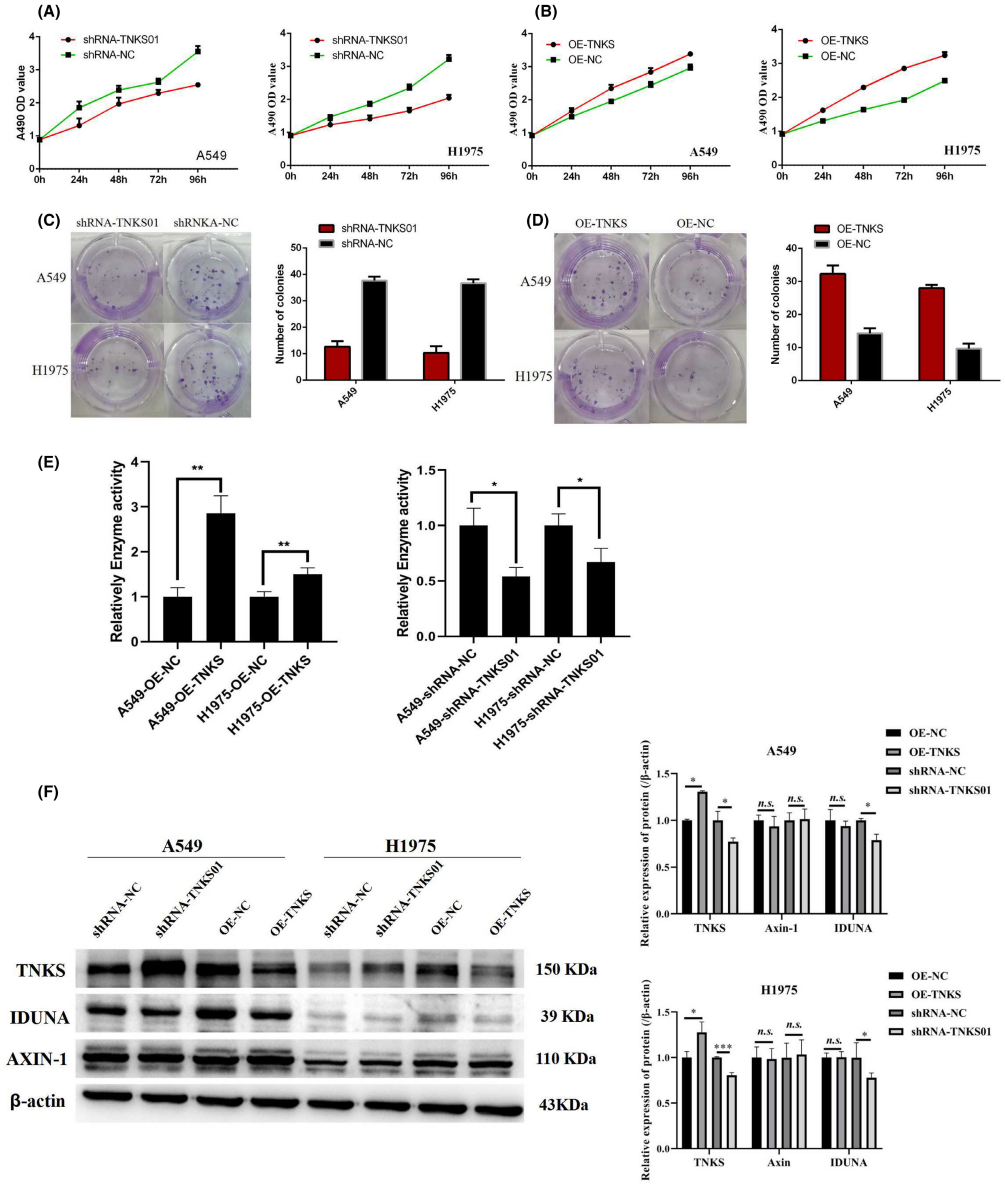
TNKS promoted lung adenocarcinoma cell proliferation in vitro (A and C) CCK‐8 and colony formation assays revealed that depletion of TNKS inhibited the growth and proliferation of A549 and H1975 cells. (B and D) TNKS overexpression promoted the growth and proliferation of A549 and H1975 cells. (E) PARylation assays revealed enhanced activity of TNKS‐overexpressing cells and decreased activity of TNKS‐knockdown cells in A549 and H1975 cells. (F**)**Western blot assays were adopted to determine the levels of PKA, Duplin, Stbm, β‐catenin, C‐myc, Bcl‐2 and caspase‐3 between the four groups.*p < 0.05, **p < 0.01.

The stable transfected cell lines were established, shRNAs were used to specifically knock down TNKS expression, whereas TNKS overexpression recombinant plasmid containing the full‐length coding sequence was used to increase TNKS expression. CCK‐8 and colony formation experiments showed that the reduction of TNKS inhibited the growth and proliferation of A549 and H1975 cells (Figure 2A,C), whereas overexpression of TNKS promoted the growth and proliferation of the two cell lines. PARylation assay showed enhanced activity of TNKS‐overexpressing cells and decreased activity of TNKS‐knockdown cells in A549 and H1975 cells (Figure 2D). WB analysis suggested that there was no significant difference in AXIN‐1 expression among the groups (Figure 2E), which may be due to the regulation of AXIN‐1 by other pathways such as phosphorylation. In A549 and 1975 cells, the expression of IDUNA protein in the TNKS‐knockdown group was decreased, but there was no significant difference in IDUNA expression in the TNKS‐overexpression group (Figure 2E). These results indicated that TNKS may form a complex with IDUNA and be degraded by ubiquitination.

### 3.3 High expression of TNKS promoted the migration and invasion of lung adenocarcinoma cells

**FIGURE 3:**
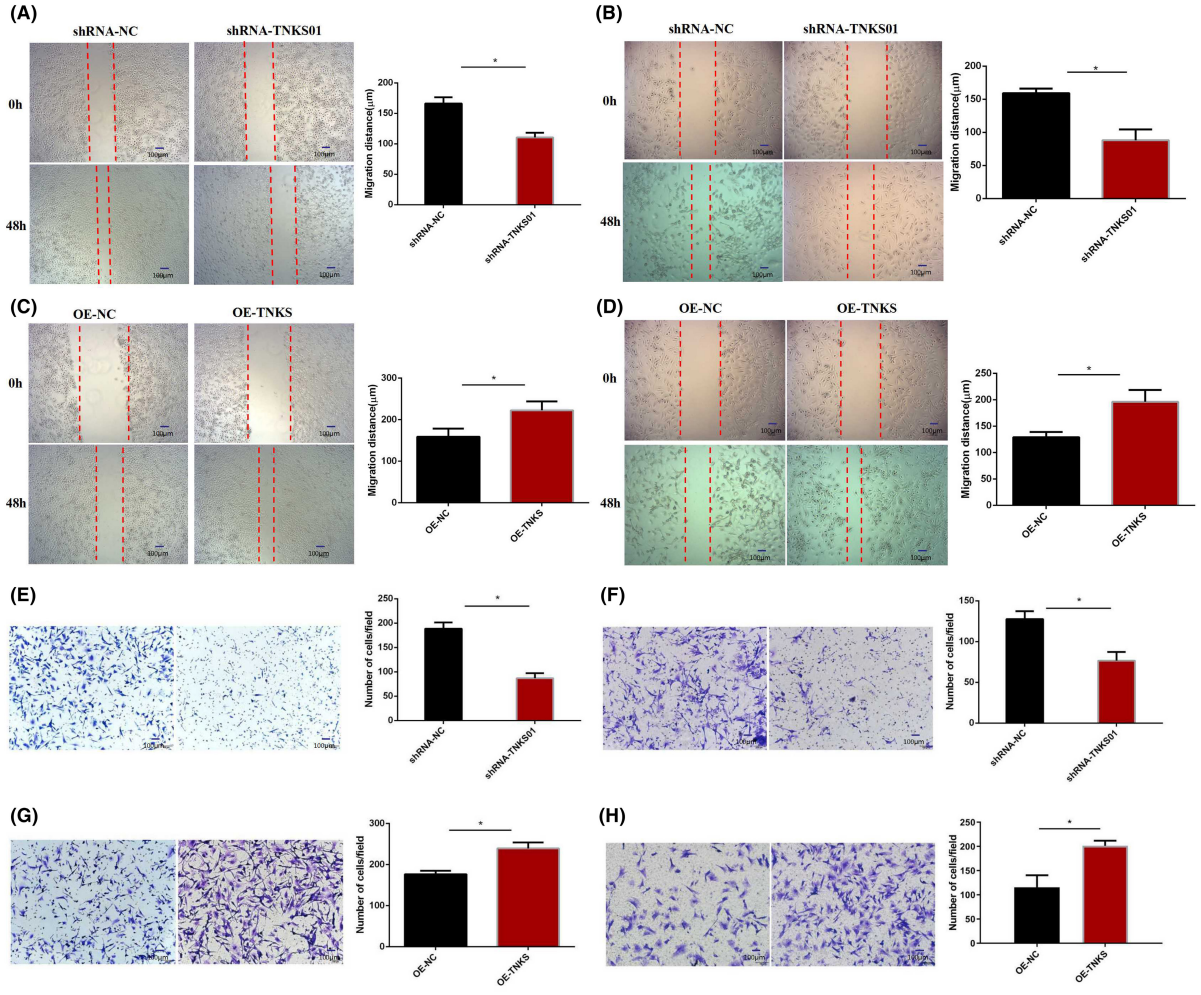
TNKS promoted the migration and invasion of lung adenocarcinoma cells. (A, B) The wound healing assay results showed that knockdown of TNKS significantly reduced the migration distance in A549 (A) and H1975 (B) cells. (C, D) TNKS overexpression significantly increased the migration distance in both cell lines. (E, F) Knockdown of TNKS significantly reduced the number of invasive A549 (E) and H1975 (F) cells. (G, H) TNKS overexpression increased the number of invasive A549 (G) and H1975 (H) cells.

Wound healing and Transwell assays were performed with A549 and H1975 cells to evaluate the effect of TNKS on the migration and invasion of lung adenocarcinoma cells. Through wound healing experiments, we found that downregulation of TNKS expression significantly reduced the migration distance of A549 (Figure 3A) and H1975 (Figure 3B) cells. In contrast, TNKS overexpression significantly raised the migration distance of the two cell lines (Figure 3C,D). Invasion assays demonstrated that knockdown of TNKS significantly decreased the number of invasive A549 (Figure 3E) and H1975 (Figure 3F) cells. In comparison, overexpression of TNKS increased the number of invasive A549 (Figure 3G) and H1975 (Figure 3H) cells. Therefore, TNKS can enhance the migration and invasion of lung adenocarcinoma cells in vitro.

### 3.4 Xiaoyan decoction and E7449 suppressed TNKS expression and inhibited adenocarcinoma cell proliferation, migration, invasion and apoptosis in vitro

**FIGURE 4:**
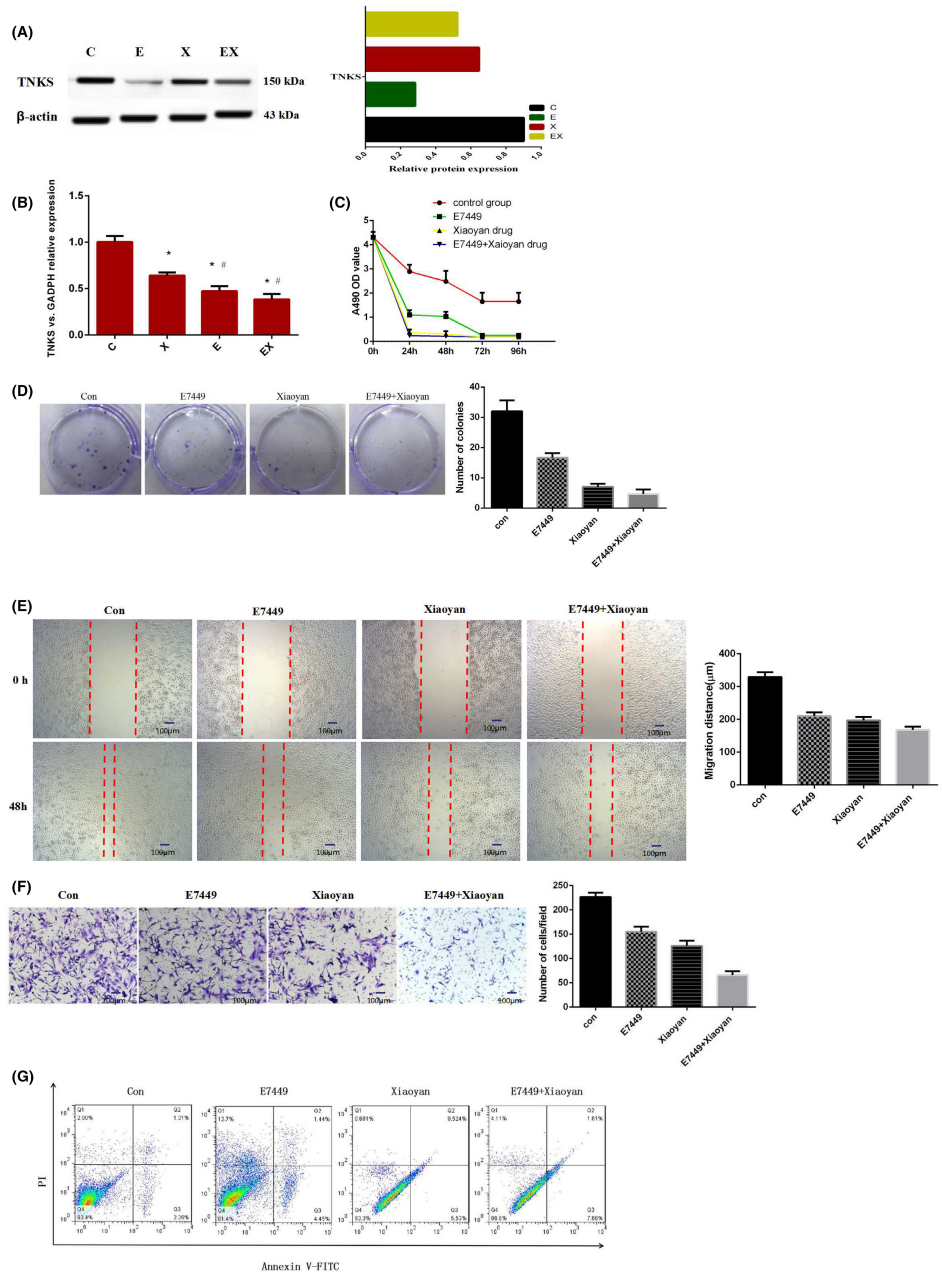
Xiaoyan decoction and E7449 inhibited A549 cell proliferation, migration, invasion and apoptosis in vitro. (A, B) Xiaoyan decoction, E7449 and the combination of both significantly downregulated the expression of TNKS. (C, D) Xiaoyan decoction and E7449 alone or in combination inhibited the growth and proliferation of A549 cells. (E, F) Xiaoyan decoction, E7449 and the combination of both significantly reduced the migration distance and the number of invasive cells compared with that in the control group. (G) Apoptosis among the groups according to annexin V‐FITC/PI double staining.

A549 cells were treated with different drugs for clinical purposes, including the control group (9% NaCl), Xiaoyan decoction group, E7449 group and Xiaoyan decoction plus E7449 group. Xiaoyan decoction, a small molecule inhibitor E7449 and Xiaoyan decoction combined with E7449 significantly downregulated the expression of TNKS (Figure 4A,B). Xiaoyan decoction and E7449 alone or in combination inhibited the growth and proliferation of A549 cells, as shown by CCK‐8 and colony formation assays (Figure 4C,D). The wound healing and invasion assay results showed that, compared with the control treatment, Xiaoyan decoction, E7449 and the combination of both significantly reduced the migration distance and the number of invasive cells (Figure 4E,F). Compared with that in the control group, the early apoptosis rate of the Xiaoyan decoction group, E7449 group and combined group was significantly greater according to annexin V‐FITC/PI double staining (Figure 4G). The results showed that early apoptosis gradually increased obviously after treatment with E7449, Xiaoyan decoction or the combination treatment. These experimental results suggested that Xiaoyan decoction and E7449 inhibited A549 cell proliferation, migration, and invasion and induced early apoptosis.

### 3.5 Drugs inhibit tumorigenesis through the Wnt pathway

**FIGURE 5:**
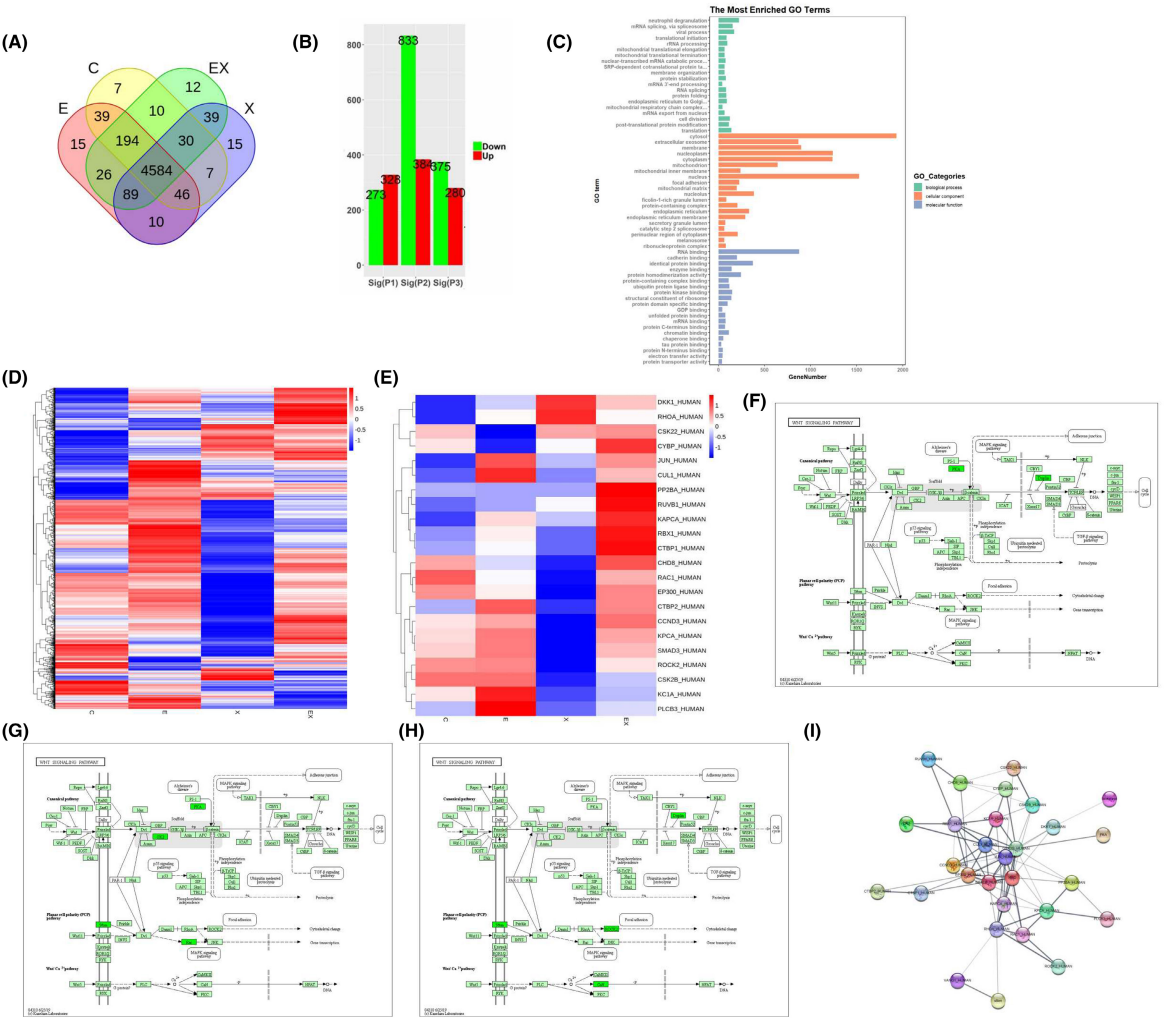
Effects of drug treatment on the protein expression profiles of A549 cells. (A) Venn diagrams revealed a total of 5123 proteins and the differentially expressed proteins in the control group, E7449 group, Xiaoyan decoction group, and Xiaoyan plus E7449 group were 7, 15, 15 and 12, respectively. Furthermore, 4584 differentially expressed proteins coexisted in all groups (A). (B) There were 384, 328 and 280 proteins upregulated in the Xiaoyan decoction, E7449 and combined groups (indicated as X, E and EX in the figure) compared with the control group, respectively. In contrast, 833, 273 and 375 proteins were downregulated in the Xiaoyan decoction, E7449 and combined groups, respectively. (C) GO analysis of the related genes showed that the common differentially expressed genes were associated with the top 20 cellular components, molecular functions, and biological processes. (D) A heatmap was generated for the principal component analysis of all the proteins. (E) A heatmap was generated for the DEPs involved in the WNT signalling pathway. (F, G and H) KEGG analysis showed downregulated proteins in the Wnt pathway in each administration group compared to those in the control group. (I) PPI analysis revealed that TNKS, PKA, Duplin and stbm closely interact with other proteins and corresponded to critical nodes in the network.

To elucidate the mechanism underlying tumour suppression of TNKS‐mediated drug, A549 cells were treated with 9% NaCl (as a control group), Xiaoyan decoction, E7449 or Xiaoyan unit E7449 for 48 h, after which the proteins were harvested, the changes in the proteins were assessed via label‐free profiling. Venn diagrams revealed that the number of differentially expressed proteins in the control group, E7449, Xiaoyan decoction, and Xiaoyan plus E7449 groups were 7,15, 15, and 12, respectively (Figure 5A). Furthermore, 4584 differentially expressed proteins coexisted among the groups (Figure 5A). Compared with those in the control group, the column diagrams revealed 384, 328 and 280 differentially upregulated proteins in the Xiaoyan decoction, E7449, and Xiaoyan plus E7449 groups, respectively. In contrast, there were 833, 273, and 375 downregulated proteins in the Xiaoyan decoction, E7449 and Xiaoyan plus E7449 groups, respectively (Figure 5B). GO analysis showed the top 20 cellular components, molecular functions, and biological processes for the genes related to the common differentially expressed proteins (Figure 5C). Next, a heatmap was generated for principal component analysis of all the proteins (Figure 5D). Heatmap analyses revealed that genes whose expression changed in the Xiaoyan decoction group was more prominently downregulated than that in the other treatment groups (Figure 5D). The significantly downregulated proteins in each treatment group were involved mainly in WNT signalling pathways (Figure 5E). KEGG analysis of the Wnt pathway revealed downregulated proteins in each treatment group compared to those in the control group (Figure 5F–H). The PKA and Duplin proteins associated with the Wnt/β‐catenin pathway were significantly downregulated in the E7449 group (Figure 5F), while the Stbm and Rac proteins associated with the WNT PLAN pathway were significantly downregulated in the Xiaoyan decoction group (Figure 5G). PPI network analysis revealed that TNKS, PKA, Duplin, stbm and Jun closely interacted with other proteins and corresponded to critical nodes in the network (Figure 5I). These data indicated that E7449 treatment may be most closely associated with the classic Wnt/β‐catenin signalling pathway, whereas Xiaoyan decoction treatment may be most closely related to the WNT PLAN pathway.

### 3.6 Validation of label‐free selected proteins by Western blot

**FIGURE 6:**
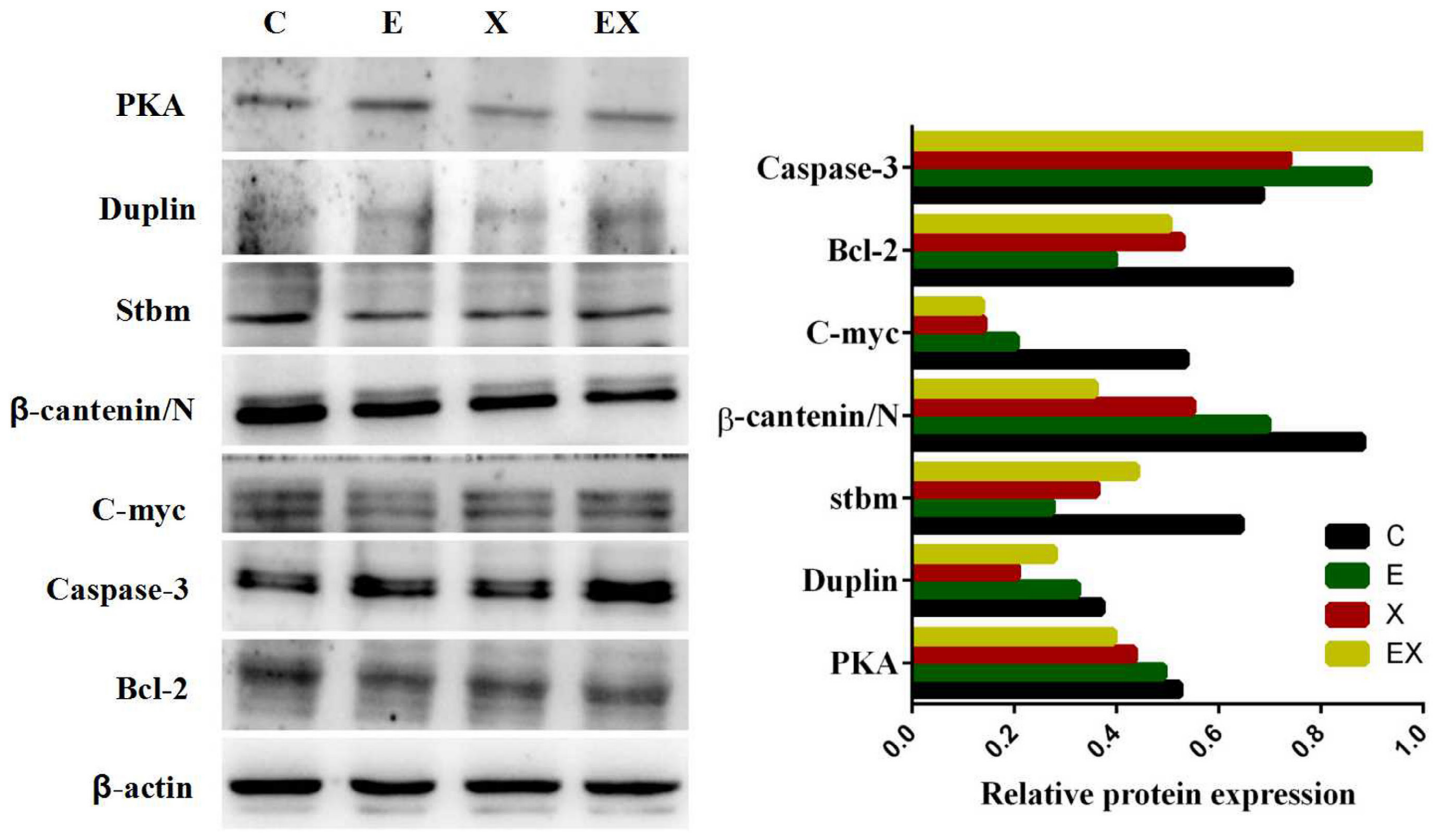
Western blot assays were adopted to compare the levels of PKA, Duplin, Stbm, β‐catenin, C‐myc, Bcl‐2 and caspase‐3 among the four groups.

To further verify the proteins identified via proteomic analysis and the associated downstream proteins, Western blot assay was adopted to determine the levels of PKA, Duplin, Stbm, β‐catenin, C‐myc, Bcl‐2 and caspase‐3 among the four groups. The results showed that the protein expression levels of PKA, Duplin and Stbm were lower in the three other groups than in the control group, which were consistent with the proteomics analysis (Figure 6). Western blotting (Figure 6) demonstrated that the level of β‐catenin, C‐myc, and Bcl‐2 protein expression in E7449 group, Xiaoyan decoction group and Xiaoyan plus E7449 group were significantly reduced compared with the control group, whereras the expression of caspase‐3 is precisely reversed.

### 3.7 Treatment with E7449 or Xiaoyan decoction inhibited lung tumour growth in vivo and attenuated the Wnt signalling pathway in adenocarcinoma

**FIGURE 7:**
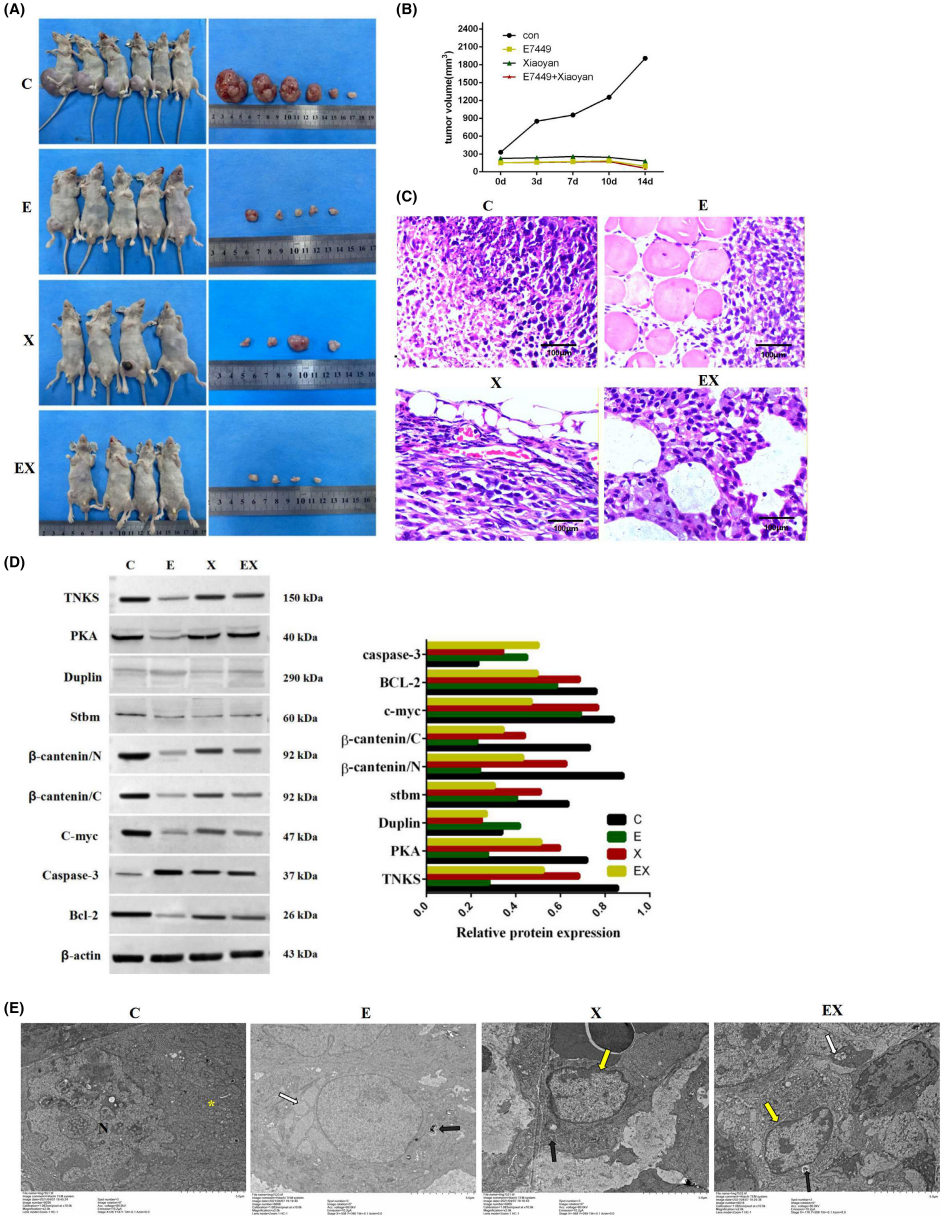
E7449 or Xiaoyan decoction inhibited lung tumour growth in vivo by regulating the Wnt signalling pathway. (A) Subcutaneous tumour formation experiments in nude mice treated with NaCl (*n* = 6), E7449 (*n* = 5), Xiaoyan decoction (*n* = 4) and E7449 joint Xiaoyan decoction (*n* = 4). Representative images of xenograft tumours are shown in the right panel. (B) Tumour volume in the xenograft mice from the treatment group and the control group. (C) Haematoxylin and eosin staining showed that compared to those in the control group, the malignant and aggressive abilities of the E7449 or Xiaoyan decoction group were markedly attenuated. (D) PKA, Duplin, Stbm, β‐catenin, C‐myc, and Bcl‐2 expression was downregulated and caspase‐3 expression was upregulated compared to that in the control group. (E) Electron microscopy image showed ultrastructural alterations in each group. “N” represents the nucleus of the tumour cells in the control group, which were large and irregular. Yellow “*” indicated that the mitochondria were mildly enlarged. In E7449 group, the black arrow shows the cellular autophagy and the white arrow shows that endoplasmic reticulum swelling was observed in the cytoplasm. In Xiaoyan decoction group, the yellow arrow indicated that the chromatin had a tendency of edge aggregation and the black arrow showed mitochondrial autophagy. Furthermore, the yellow arrow indicated that the chromatin was condensed and that edge aggregation occurred; autophagy (black arrow) and mitochondrial swelling (white arrow) were found in the cytoplasm of the group treated with E7449 combined with Xiaoyan decoction group.

Subcutaneous tumour formation experiments revealed that the tumour size was significantly reduced in the E7449 group and Xiaoyan decoction group, especially in the combined group. It illustrated that E7449 or Xiaoyan decoction significantly inhibited the formation of subcutaneous tumours from lung adenocarcinoma cells in vivo. (Figure 7A,B). Compared to those in the E7449 or Xiaoyan decoction group, the malignant and aggressive abilities of the control group were markedly attenuated according to haematoxylin and eosin staining (Figure 7C). In addition, Western blot analysis showed that PKA, Duplin, Stbm, β‐catenin, C‐myc, and bcl‐2 were downregulated and caspase‐3 was upregulated compared to the control group (Figure 7D). β‐catenin is an important regulatory protein of the WNT pathway, and the c‐myc protein is a transcript encoded by a proto‐oncogene downstream of the WNT pathway. Both bcl‐2 and caspase‐3 are closely related to apoptosis. These results indicated that E7449 or Xiaoyan decoction prevented lung tumour growth and induced apoptosis in vivo by attenuating the Wnt pathway. The results of electron microscopy results (Figure 7E) showed that the tumour cells in the control group were abundant in number, irregular in shape, complete in cell membrane, evenly distributed in organelles, large and irregular in nucleus, and had no apoptotic bodies. In E7449 group, the number of tumour cells decreased and mitochondrial autophagy was observed in the cytoplasm. In Xiaoyan decoction group, the number of tumour cells decreased, the cells became smaller, the cell membrane was broken, the intracytoplasmic organelles were destroyed, and the chromatin tended to aggregate at the edge. In E7449 combined with Xiaoyan decoction group, the number of tumour cells was significantly reduced, the cell membrane was completely broken, a large number of organelles were destroyed, the chromatin was condensed and appeard edge aggregation. Electron microscopy revealed that mitochondrial autophagy, chromatin edge sets and other early apoptotic processes occurred to different degrees in all the drug administration groups compared with those in the control group.

## 4 DISCUSSION

TNKS is a member of the PARP protein superfamily, including two subtypes, TNKS 1 and TNKS 2, which can catalyse protein substrates by covalent posttranslational modification. TNKS1 and TNKS2 are also known as PARP5a and PARP5b, respectively.Qiu et al have been developed as inhibitors for tumours with elevated β‐catenin activity.^18^, ^19^, ^20^ Studies have found that TNKS1/2 can positively regulate the abnormal activation of WNT pathways in tumours.^21^ Repressing TNKS activity through either genetic or pharmacological approaches antagonized canonical Wnt signalling, reduced murine and human lung cancer cell line growth, and decreased tumour formation in mouse models.^22^ TNKS plays an important role in the WNT pathway by directly binding to Axin.^23^, ^24^ In our study, we first explored the relationship between TNKS and clinicopathological features. TNKS was highly expressed in lung adenocarcinoma tissues. Furthermore, the high TNKS expression in 74 lung adenocarcinoma patients was closely related to large tumour size and advanced TNM stage. The biological function of TNKS revealed that depletion of TNKS inhibited the growth and proliferation and reduced the migration and invasion of A549 and H1975 cells. TNKS overexpression promoted cell growth and proliferation and enhanced the migration and invasion of lung adenocarcinoma cells in vitro. PARylation assay shows enhanced activity of overexpressed TNKS and decreased activity of knocked down TNKS in A549 and H1975 cells. These results indicate that TNKS may become a new target in the treatment of lung adenocarcinoma. According to Li et al.,^7^ tankyrase is a major upstream regulator of the LKB1‐AMPK pathway and provids another focus for cancer and metabolic disease therapies. TNKS inhibition or knockdown reduced ovarian cancer cell proliferation, colony formation, migration, invasion, and tumorigenic potential in nude mice, and these changes correlated with the downregulation of targets (cyclin D1, MDR, and MMP‐9) of Wnt/β‐catenin signalling.^6^ TNKS inhibitors can control WNT hyperactivation and provide long‐term tumour control in vivo.^21^ Our previous study^25^ found that XAV939, a small molecule inhibitor of TNKS, could inhibit the proliferation of lung adenocarcinoma A549 cells by downregulating the Wnt pathway. In addition, we found that the expression of TNKS protein was significantly downregulated after Xiaoyan decoction was administered to lung adenocarcinoma cells. According to the clinical experience of tumour treatment, the principle of ‘strengthening, detoxifying and removing blood stasis’ was proposed for this purpose. Xiaoyan decoction has been used in clinical antitumour therapy practice for 15 years because it has strong antitumour activity.^26^, ^27^, ^28^, ^29^ Xiaoyan decoction and a small molecule inhibitor both act on TNKS proteins and are new candidates for the treatment of lung adenocarcinoma. If both drugs are applied together, will their effects be synergistic? On this basis, we assessed the anticancer effect and possible mechanism of TNKS in lung adenocarcinoma cells by the synergistic use of a TNKS small molecule inhibitor and the TCM Xiaoyan decoction, of which TNKS is a key target, and we discussed the idea of integrating Chinese and Western medicine. Our study confirmed that Xiaoyan decoction and the small molecule inhibitor E7449 have the biological characteristics of inhibiting A549 lung adenocarcinoma cell proliferation, migration and metastasis in vivo and in vitro. In addition, the two agents were more effective in combination. Furthermore, treatment with E7449 or Xiaoyan decoction significantly inhibited the formation of subcutaneous lung adenocarcinoma tumours in vivo. The malignant and aggressive abilities of the E7449 or Xiaoyan decoction group were markedly attenuated compared to the control group. To elucidate the mechanism underlying the effect of Xiaoyan decoction and E7449 on lung adenocarcinoma, label‐free proteomics was conducted. Proteomics research showed that the expression of PKA, Duplin and stbm, which are related to the WNT pathway, were significantly changed in the Xiaoyan decoction or E7449 group relative to the control group. The results of the proteomics analysis showed that most of the proteins in the E7449 group were associated with the classic Wnt/β‐catenin signalling pathway, whereas those in the Xiaoyan decoction group were mostly associated with the WNT PLAN pathway. Numerous studies have shown that the constitutive components of Wnt signalling are altered in breast cancer cells. These alterations include mutations, amplifications, deletions, and methylations that occur at the DNA level, posttranscriptional modifications that occur at the mRNA level, and posttranslational modifications that occur at the protein level.^30^, ^31^ In cutaneous melanoma, nuclear β‐catenin binds to TCF/LEF‐type transcription factors and consequently stimulates the expression of downstream genes, such as cyclin D1 and c‐myc. Overexpression of these genes alter cell cycle progression and contributes to tumorigenesis.^32^ Research has shown that the Wnt/PLAN pathway is associated with tumour cell metastasis and invasion.^33^ It was revealed that activating the WNT/planar cell polarity (PCP) signalling pathway can promote metastasis in colorectal cancer.^34^ Vorst et al.^35^ hypothesized that the engagement of the Wnt/PCP pathway after tumour initiation drives malignancy by promoting cellular proliferation and invasion and that the ability of Wnt/PCP signalling to supplant oncogene addiction may contribute to tumour resistance to oncogenic pathway‐directed therapeutic agents. In this study, regulatory proteins of the classical Wnt/β‐catenin pathway and their downstream proteins, including β‐catenin, PKA, Duplin, c‐myc, Bcl‐2 and caspase‐3, were verified in A549 cells and mice. The regulatory proteins Stbm and Jun are involved in the non‐classical Wnt/PLAN pathway. We preliminarily explored the possible mechanism by which Xiaoyan decoction inhibits cell proliferation by inhibiting the classical Wnt/β‐catenin pathway and non‐classical Wnt/PLAN pathway. According to the results of the label‐free profiling and Western blotting results, we speculated that on the one hand, the nuclear entry of β‐catenin could be prevented by downregulating TNKS to reduce the activity of the Wnt pathway; on the other hand, the proliferation of lung adenocarcinoma cells could be inhibited by the Wnt/PLAN pathway. Furthermore, the downregulation of c‐myc and β‐catenin/N was the most obvious in the E7449 group, which further indicated that the small molecule inhibitor E7449 may regulate the classical WNT/β‐catenin classical pathway. WB detection showed that the E7449 group, Xiaoyan decoction and combined treatment group could regulate the apoptosis‐related proteins Bcl‐2 and caspase‐3, indicating that tumour cell apoptosis was promoted by E7449 or Xiaoyan decoction. By inhibiting the Wnt pathway, the expression of the downstream target protein c‐myc was further downregulated, and apoptosis was promoted via the regulation of the apoptosis‐related proteins Bcl‐2 and caspase‐3. However, the specific action mechanism of each pathway and the interactions between the pathways after the regulation of Xiaoyan decoction need to be further studied. In summary, our results provide a basis for the study of the molecular mechanism by which Xiaoyan decoction regulates the signal transduction pathway of lung adenocarcinoma cells and provide a theoretical basis for the clinical treatment of lung adenocarcinoma with TCM and the combination of traditional Chinese medicine and Western medicine. However, further research is needed to elucidate the specific underlying mechanism involved.

## AUTHOR CONTRIBUTIONS

**Xu Zheng:** Conceptualization (lead); project administration (equal); writing – original draft (lead); writing – review and editing (equal). **Yanyan Han:** Data curation (equal); resources (equal). **Lili Gu:** Data curation (equal); software (equal). **Shan Gao:** Methodology (equal); supervision (equal). **Yan Lv:** Formal analysis (equal); methodology (equal). **Chong Li:** Funding acquisition (lead); investigation (equal); project administration (equal); supervision (equal).

## FUNDING INFORMATION

The present study was supported by the National Natural Science Foundation of China (grant no. 81774054), the Scientific Research Project of Tianjin Municipal Education Commission, China (grant no.2018KJ038), the Scientific Research Project of Tianjin Municipal Education Commission, China (No. 2023KJ150) and Extension project of national natural science foundation incubation of First Teaching Hospital of Tianjin University of Traditional Chinese Medicine, China (No. 2023005).

## CONFLICT OF INTEREST STATEMENT

The authors confirm that there are no conflicts of interest.

## Supporting information


Figure S1.

Table S1.

## Data Availability

The data that support the findings of this study are available from the corresponding author upon reasonable request.
