# Supplementary figures and images for: Study of the mechanism by which Xiaoyan decoction combined with E7449 regulates tumorigenesis in lung adenocarcinoma
Source: J Cell Mol Med. 2024 Jun 19;28(12):e18467. doi: 10.1111/jcmm.18467 (PMC11186742; doi:10.1111/jcmm.18467)

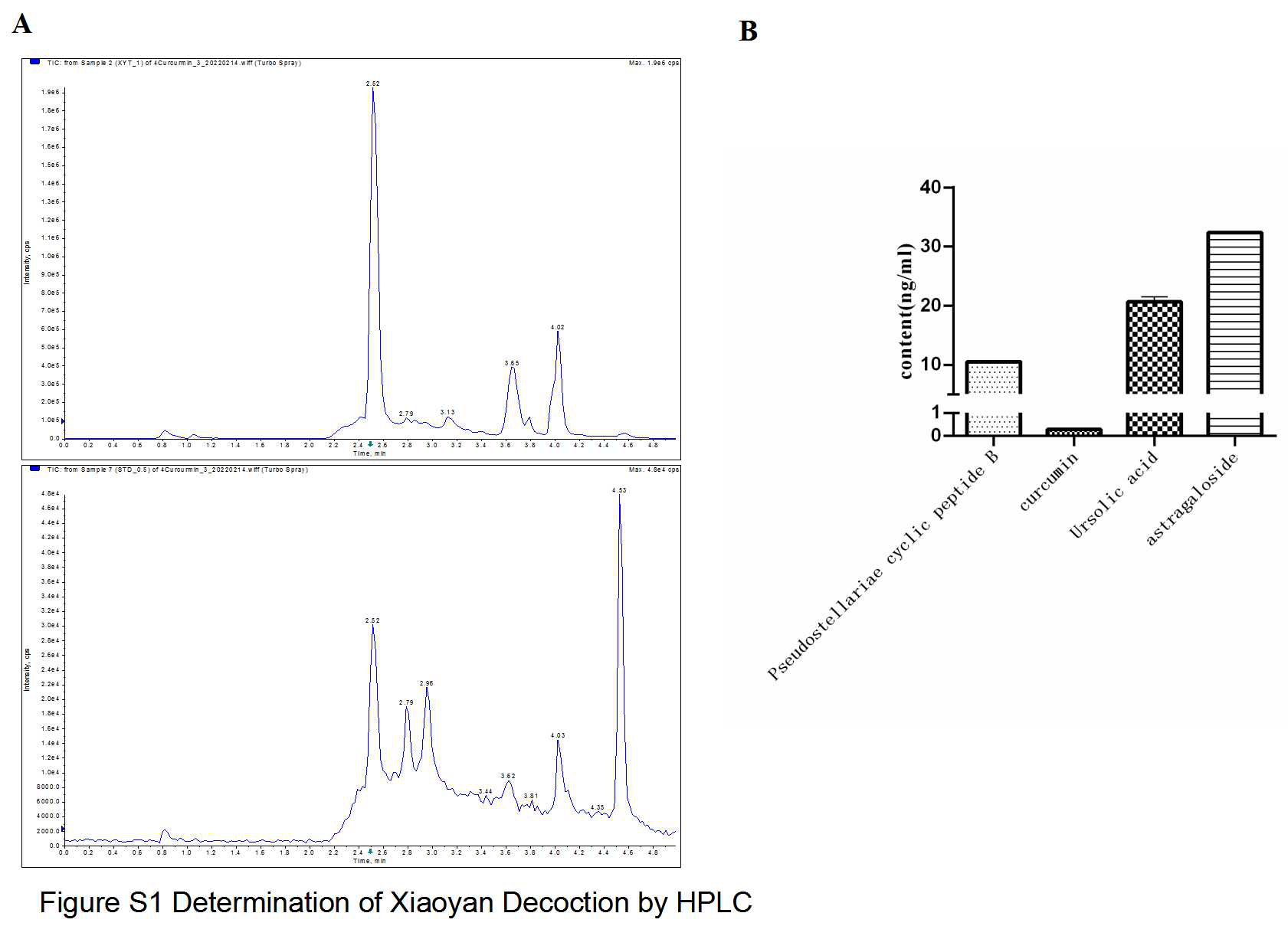

Supplement: Supplementary file 1 — Figure S1. [file JCMM-28-e18467-s001.tiff]
