# Supplementary material for: Study of the mechanism by which Xiaoyan decoction combined with E7449 regulates tumorigenesis in lung adenocarcinoma
Source: J Cell Mol Med. 2024 Jun 19;28(12):e18467. doi: 10.1111/jcmm.18467 (PMC11186742; doi:10.1111/jcmm.18467)

**Appendices**

| Table S1. Polymerase chain reaction primers used in this study. | |
| --- | --- |
| Gene | Sequence, 5'-3' |
| tankyrase | Forward CTGGCAGATCCTTCAGCAAA |
|  | Reverse CCCATCACTTGCATGGCAAT |
| GAPDH | Forward GGCATCCTGGGCTACACTGA |
|  | Reverse GAGTGGGTGTCGCTGTTGAA |
| annotation: Primerswas synthesized by Huzhou Hippo Biotechnology Co., Ltd. | |

| TableS2 shRNA sequence and Vehicle components sequence | |
| --- | --- |
| Insert content | shRNA sequence |
| TNKS shRNA01 | 5’-GCGAAAGTCGACTCCTTTACA CGAATGTAAAGGAGTCGACTTTCGC-3’ |
| Neg control shRNA | 5’-CCTAAGGTTAAGTCGCCCTCGC CGAAGCGAGGGCGACTTAACCTTAGG-3’ |
|  |  |
|  | Vehicle components sequence |
| OE-TNKS | CMV-MCS-IRES-PURO |
| NC-TNKS | CMV-MCS-3flag-IRES-Puro |
| annotation: The above sequence was synthesized by Tianjin Sheweisi Biotech Co.,Ltd. | |


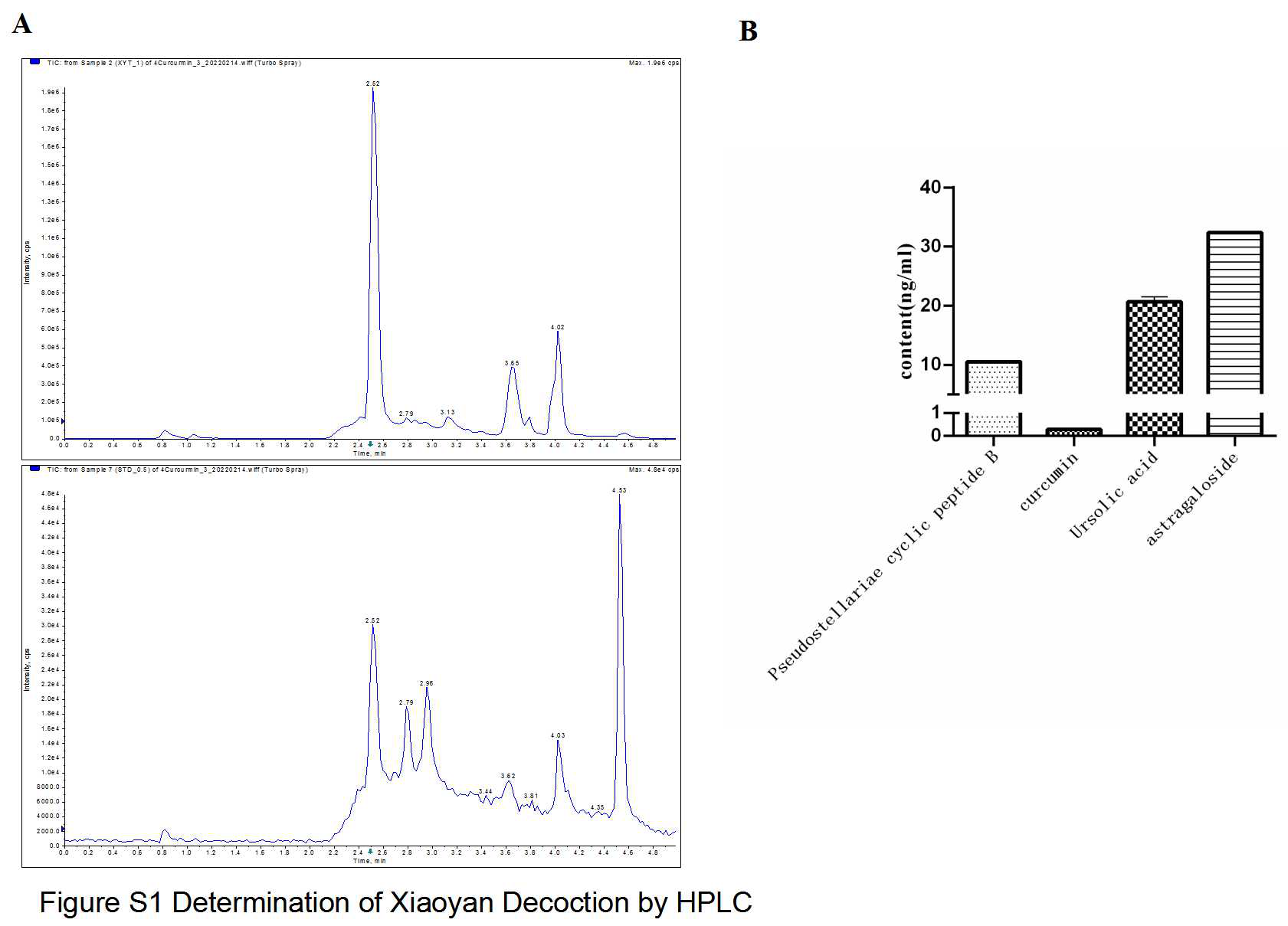

Supplement: Supplementary file 2 — Table S1. [file JCMM-28-e18467-s002.docx]
